# A Review on the Valorization of Recycled Glass Fiber-Reinforced Polymer (rGFRP) in Mortar and Concrete: A Sustainable Alternative to Landfilling

**DOI:** 10.3390/polym17192664

**Published:** 2025-10-01

**Authors:** Mohamed Wendlassida Kaboré, Didier Perrin, Rachida Idir, Patrick Ienny, Éric Garcia-Diaz, Youssef El Bitouri

**Affiliations:** 1 Laboratoire Mécanique et Génie Civil (LMGC), IMT Mines Ales, University Montpellier, CNRS, F-30100 Ales, France; wendlassida.kabore@mines-ales.fr (M.W.K.); patrick.ienny@mines-ales.fr (P.I.); eric.garcia-diaz@mines-ales.fr (É.G.-D.); 2 PCH, IMT Mines Ales, F-30100 Ales, France; didier.perrin@mines-ales.fr; 3 Cerema, Gustave Eiffel University, UMR MCD, F-77171 Sourdun, France; rachida.idir@cerema.fr

**Keywords:** recycled glass fiber-reinforced polymer (rGFRP), recycling, concrete, mechanical strength

## Abstract

The recycling of glass fiber-reinforced polymer (GFRP) in cementitious materials is an interesting way of managing the end of life of this type of material. As the solutions of landfilling and incinerating have reached their limits, the material recovery by recycling approach appears to be suitable to develop cement-based materials with enhanced properties. Different recycling methods, including mechanical, thermal and chemical recycling, are commonly used for the recovery of fibers and resins. Mechanical recycling is more suitable due to its low cost and ease of implementation. Moreover, mechanical recycling has limited environmental impact and is ideal for use with cementitious materials (fiber and resin). Several studies are being conducted to find the best incorporation method, notably the incorporation of recycled GFRP of different sizes (small, medium, large and coarse) and shapes (fibrous, cubic, random) as a substitute for sand and/or aggregate in mortars and concretes or as reinforcement materials. This article aims to establish a state of the art perspective on the incorporation of rGFRP into cement-based materials. The benefits of this incorporation are highlighted as well as the limitations. The various challenges to be overcome to make this incorporation useful from a practical point of view are reported.

## 1. Introduction

Glass fiber-reinforced polymers (GFRPs) exhibit a synergistic combination of properties that surpass those of the individual constituents, as neither the glass fibers nor the polymer matrix alone can achieve the enhanced mechanical performance observed in the composite material [1]. Their excellent properties (lightness, resistance, durability) allow them to be widely used [1]. Depending on the matrix, there are different types of glass fiber composites, such as thermosets and thermoplastics. The matrix is either a thermosetting resin (unsaturated polyester, epoxy, vinylester, phenolic, polyimide, polyurethane, etc.) or a thermoplastic resin (polyamide, polycarbonate, saturated polyester, polypropylene, etc.). Thermoplastic resins are supplied as solid aggregates that are heated until softened or melted, allowing them to be shaped and combined with glass fibers. In contrast, thermosetting resins are liquid precursors that react with catalysts to form a rigid matrix through chemical curing. These two types of matrices differ in their processing methods: thermoplastics are molded after melting, whereas thermosets consist of liquid reagents that impregnate the fiber preform at room temperature. These differences strongly influence the manufacturing process and later recyclability of GFRP composites. The anisotropic properties of the fibers, combined with their tailored composition, enable the composite material to rival steel in performance, despite having only one-quarter of its specific weight, while also exhibiting higher stiffness.

Due to their high mechanical performance, fiber-reinforced polymers are used in several engineering applications. GFRPs are widely used in the marine industry to manufacture the hulls and decks of pleasure boats (yachts, jet skis, sailboats) for their corrosion resistance, light weight and good reinforcing effect, giving these vessels exceptional longevity. Furthermore, and thanks to their toughness, impact resistance and flame-retardant (when to treat with additives) properties, these materials are used in the automotive, aerospace, military and even sports sectors. GFRPs are used in the electrical and electronics industries for their insulating and corrosion-resistant properties (insulators, insulating tools, electrical junction boxes, dashboard covers). Beyond these industrial sectors, GFRPs are widely present in everyday materials due to their versatility and performance. In residential applications, GFRPs are widely used in the manufacturing of furniture, bathtubs, shower trays and spas. In the leisure industry, they are employed in the construction of attractions such as carousels, towers, themed castles, water slides, Jacuzzis and other recreational structures. Their low porosity, non-staining and wear-resistant finish also allows their use in the medical field (e.g., instrument housings for X-ray beds).

Due to their widespread use, significant efforts are made to ensure the production and the availability of the primary materials (glass fiber and resin). Glass fibers are derived from mineral-based raw materials, while the broad variety of available resins represents a significant advantage, as they can be formulated in both thermoplastic and thermosetting forms. GFRP production has been growing steadily due to the strong demand in the transportation, electrical, electronics, wind energy, pipeline and tank sectors; an annual growth rate of 6.4% was recorded between 2017 and 2022 [2]. Glass fiber alone was valued at USD 28.7 billion in 2024 (with E-glass accounting for 65% of the market), with projections estimating a market value of USD 54 billion by 2034.

**Figure 1:**
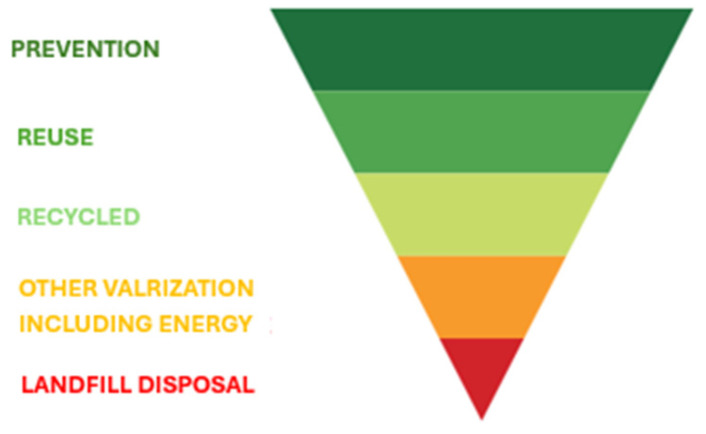
Waste management hierarchy.

Global production of glass fiber composites, which exceeds 10 Mt/year [3], generates significant volumes of end-of-life GFRP, posing an increasing challenge for their management. European Directive 2008/98/EC establishes a regulatory framework based on a waste management hierarchy that prioritizes prevention, reuse, and recycling, while relegating landfilling and incineration to last-resort solutions (Figure 1). More than 90% of all fiber-reinforced composites produced are GFRPs [3,4], nearly 60% of which are thermoset-based polymers that are difficult to recycle because they cannot be remelted once cured. In the absence of viable alternatives, most of these materials are still disposed of through landfilling or incineration [5,6], despite the restrictions imposed by Directive 1999/31/EC on large composite parts [7,8]. Recycling GFRP therefore remains a major challenge, primarily due to the difficulty of separating and recovering glass fibers from the polymer matrix [6,7].

This review aims to provide a comprehensive overview of the recycling and reuse of glass fiber-reinforced polymers (GFRPs) in cementitious materials. It first outlines the main recycling technologies currently available for GFRP waste, including mechanical, thermal, and chemical processes, highlighting their advantages and limitations.

Subsequently, this study reviews recent research on the incorporation of recycled glass fiber-reinforced polymers (rGFRPs) into mortars and concretes, with a particular focus on their influence on both fresh and hardened properties, including workability, mechanical strength, toughness, density, and durability-related performance.

Finally, potential applications and perspectives on the use of rGFRP in construction are discussed, identifying opportunities and research needs for improving its valorization.

## 2. GFRP Recycling Techniques

Different recycling methods have been developed, including mechanical, thermal and chemical recycling. The choice of recycling technology depends on several factors, namely the nature of the material matrix (thermoplastic or thermosetting) and the intended application.

### 2.1. Mechanical Recycling

**Figure 2:**
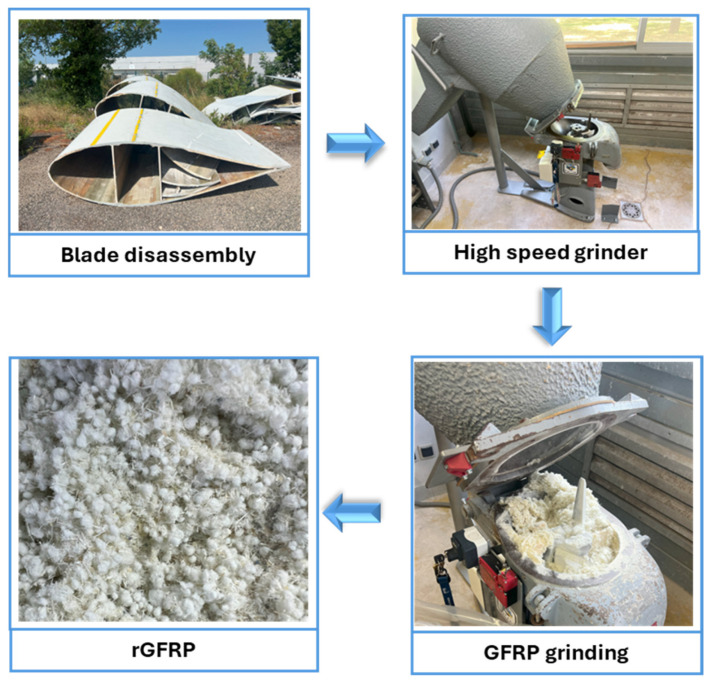
Schematic diagram of mechanical grinding.

Mechanical recycling consists of reducing GFRP into smaller pieces (Figure 2). The material is crushed using processes such as grinding, crushing, shredding or similar techniques to obtain small-sized particles. Depending on the reuse application, sieving can be performed after grinding to select suitable fractions. Recycled materials can thus be produced in varying sizes and shapes depending on their final use [8]. Mechanical recycling is often used as a preliminary step before other recycling techniques. For large parts, it is typically performed before thermal or chemical treatments [8]. Downsizing GFRP elements in this manner represents a crucial step that facilitates their reuse in subsequent applications. Thermoset composite pieces can even be reused in the production of thermoplastic composites [9,10,11]. However, it should be noted that mechanical recycling generally results in a loss of material performance.

The mechanical recycling process appears to allow control over the size, shape and fiber/resin fraction recovered. Palmer et al. [9] ground GFRP in a grinder equipped with an 8 mm diameter screen (which they considered to be the process that produces longer fiber fractions), then separated the recycled particles according to size, shape and density using a cascade air classification or zigzag air classifier. They obtained rGFRP composed of 70% coarse and fibrous fractions and 30% fine particles (powder). Kouparitsas et al. [11] carried out a grinding process (two grinding stages with the same grinder) and screening in a mini-granulator equipped with two parallel grinding discs on GFRP. In the grinder, this consists of a continuous and rapid grinding process (material residence time less than 2 s). It was found that the second grinding had no effect on the size of the recyclates, and the recovered materials had fiber-rich fractions (glass fiber lengths between 4 and 7 mm) and fine fractions. Bream et al. [10] carried out two stages of grinding GFRP in grinders equipped with screens of different sizes (20 mm and 4 mm). They observed that the materials retained their primary integrity, namely bundles of fibrous particles, most of which were encapsulated in the resin matrix, with 69% of the recycled material having a size greater than 1 mm.

### 2.2. Thermal Recycling

It mainly consists of thermal degradation of composites at high temperatures, breaking the bonds between the fibers and the resin matrix. At elevated temperatures, the resin burns or evaporates, leaving behind the fibers [12]. The treatment temperature is a critical factor, as it strongly influences the quality of the recovered fibers [8,13]. If the temperature is too low, carbonized residues remain on the fiber surface, whereas excessive temperatures can reduce fiber diameter [12,14,15] (at very high temperatures, potentially oxidation if the atmosphere is not inert in the case of carbon fiber, and molecular restructuring or partial melting in the case of glass fiber). The mechanical properties of the fibers are also affected, with their performance decreasing as the treatment temperature increases [16]. Thermal recycling is commonly used for the recovery of both glass and carbon fibers [17]. In practice, the composites are incinerated, and three main types of thermal processes are used to recover the fibers: pyrolysis, fluidized bed treatment, and microwave-assisted treatment.

- Pyrolysis

**Figure 3:**
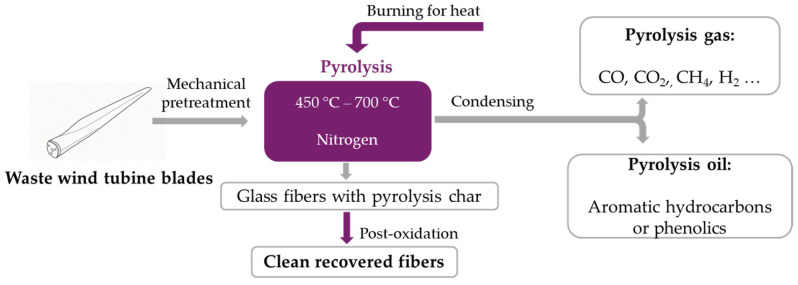
Procedure for pyrolysis recycling of waste wind turbine blades (adapted from [16]).

It is one of the most commonly used heat treatments for fiber-reinforced polymers (including GFRP) and is carried out under a nitrogen atmosphere, with no or very little oxygen. The process is generally performed at temperatures between 450 °C and 700 °C [17,18] and can reach up to 900 °C (Figure 3). Thermal decomposition during pyrolysis produces three types of products: gases (carbon monoxide, hydrogen and various hydrocarbons), liquids (oil) and solids (char and fibers) [17,18,19]. One of the main advantages of pyrolysis is its ability to recover long and relatively clean fibers [8,16]. The properties of the recovered fibers are strongly influenced by process parameters such as the heating rate, peak temperature, nitrogen (N_2_) flow rate and isothermal holding time [8]. Recycled fibers obtained through pyrolysis generally exhibit lower performance than virgin fibers [16]. Several studies report a reduction of approximately 65% in the tensile strength of recovered fibers [17,19,20,21]. However, when the process is carried out at relatively low temperatures (typically below 450 °C) [17], the fibers are able to retain up to 90% of the tensile strength of virgin fibers [8,17,19]. Analyses of the surface morphology and fiber composition, performed using scanning electron microscopy (SEM) and energy dispersive spectrometry (EDS) [17], revealed minimal surface damage and limited degradation of mechanical properties for treatment temperatures below 450 °C, whereas severe fiber degradation was observed above 600 °C [20,21].

Since 2003, several studies have sought to optimize thermal recycling processes to better preserve the quality of recycled materials. New devices and procedures primarily designed for carbon fiber-reinforced polymers (CRFPs) have been developed to prevent char formation, preserve fiber length, and improve their potential for remanufacturing [18].

- Fluidized bed (gasification)

**Figure 4:**
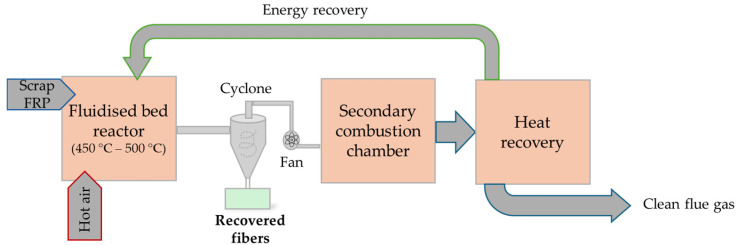
Combustion recycling process based on a fluidized bed (adapted from [16] and [22]).

Developed in the mid-1990s at the University of Nottingham, it consists of injecting a fluid (gas or liquid) through a bed of solid particles (typically silica sand) at a controlled flow rate, causing the particles to become suspended and behave like a fluid, hence the term ‘fluidized’. The waste is placed on this bed, where the polymer matrix undergoes thermal degradation due to the heat transferred from the sand. Classically, the sand is fluidized using a flow of hot air or nitrogen at temperatures between 450 °C and 550 °C and at a velocity of 0.4 to 1.0 m/s. The resin matrix evaporates, leaving behind fiber filaments and fillers, which are carried away by the airflow, while heavier components (e.g., metals, if present) remain into the bed. The fibers and fillers are then separated from the airflow by a cyclone, and the residual resin is fully oxidized in a secondary combustion chamber at 1000 °C, with the released energy recovered as heat [17,18,19]. Decomposition of fiber-reinforced polymers (FRPs) begins at around 400 °C in a fluidized bed. During gasification, chemical changes also occur on the surface of the fibers, notably the conversion of hydroxyl groups into carbonyl and carboxyl groups [8]. As with pyrolysis, higher treatment temperatures result in greater fiber degradation, with studies reporting tensile strength losses of about 50%, 65%, and 90% at 450 °C, 550 °C, and 650 °C, respectively [17,19] (Figure 4). Moreover, fibers recovered through this method are usually fluffy and dimensionally unstable, making them difficult to reuse and challenging to reintegrate into recycled composites [8].

- Microwave

This process extracts electrical energy, converts it into high-power electromagnetic waves, and channels them into the treatment compartment via a waveguide [19]. Microwave-assisted pyrolysis has proven effective for recovering both glass and carbon fibers from used composites, as it degrades or disintegrates the resin matrix into oil and gas. Its main advantage is that the material is heated internally, accelerating heat transfer, reducing peripheral heat loss, and improving energy efficiency [14]. Obunai et al. [23] tested this method under three different atmospheres (air, nitrogen, and argon) and found that processing under argon was the most effective for recovering defect-free fibers. The tensile strength of the fibers recovered using the microwave technique is nearly identical to that of virgin fibers or fibers extracted by conventional methods [17,19]. Akesson et al. [24] treated glass composites from wind turbine blades with microwaves at 300–600 °C in a nitrogen atmosphere, recovering oil and glass fibers (0–30 mm in length) with a 25% reduction in tensile strength [25]. More recently, advanced microwave-based methods have been developed to recover carbon fibers with properties surpassing those of virgin carbon fibers [26,27].

### 2.3. Chemical Recycling

**Figure 5:**
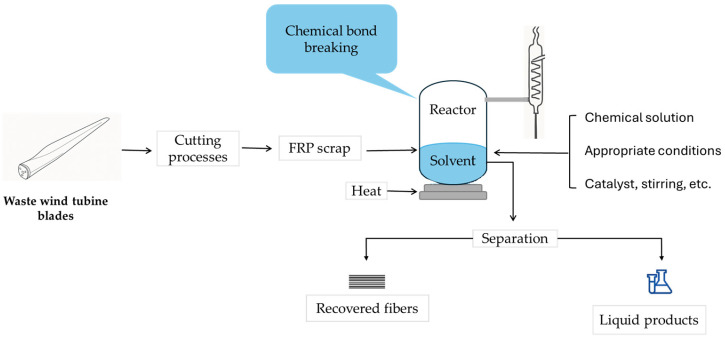
Chemical solvolysis of waste wind turbine blades (adapted from [16]).

Chemical recycling, or solvolysis, involves separating the fibers from the resin using chemical agents combined with catalysts or additives to break down the matrix bonds at specific temperatures and pressures (Figure 5). Solvolysis can be conducted under different conditions: at low temperature and pressure (LTP) or at sub- or supercritical temperatures.

- Low-temperature solvolysis (LTS)

This process is carried out below 200 °C and at atmospheric pressure, typically in an acidic medium or using solvents, such as water, alcohols, ammonia, nitric acid, sulphuric acid, or acetic acid, to break the chemical bonds between the polymer matrix and the fibers. Although low-temperature solvolysis (LTS) requires a longer reaction time, it offers better reaction control, avoids undesirable secondary reactions, and allows for the recovery of fibers with higher quality [8,19]. In subsequent carbon fiber-reinforced polymer (CFRP) recycling with LTS, the recovered fibers exhibited only a 2.9% reduction in tensile strength compared to virgin fibers [19,28].

- Sub- and supercritical temperature (solvolysis)

This process is carried out under conditions approaching or exceeding the critical temperature and pressure of the solvent. It can involve the use of water (200–450 °C) under sufficient pressure to maintain its liquid state (sub- or supercritical water solvolysis) or alcohols such as methanol, ethanol, propanol, acetone, or glycol (sub- or supercritical alcohol solvolysis) [19].

Supercritical fluids (SCFs) create a highly reactive environment that promotes the decomposition of polymer resins, enabling the recovery of high-quality fibers from GFRP and CFRP. Because SCFs are above their critical temperature and pressure, they combine gas-like diffusivity, which allows them to penetrate solids, with liquid-like solvency, enabling them to dissolve materials. This makes SCFs highly effective for digesting the resin matrix in FRP waste [17]. Studies have demonstrated that temperature and pressure are key parameters influencing the quality of glass and carbon fibers recovered through sub- or supercritical water solvolysis. Depending on the conditions, the tensile strength of the recovered fibers decreased by up to 20% compared to virgin fibers [29,30,31,32,33,34].

In the case of sub- or supercritical alcohol solvolysis, several authors reported virtually no degradation in the strength and surface properties of the recovered fibers [35,36]. Others observed tensile strengths ranging between 85% and 99% of those of virgin fibers [17,19,30,31,37]. SEM observations of carbon fibers recovered at sub- or supercritical temperatures showed clean, smooth, and defect-free surface morphologies [33,38,39,40].

Table 1 provides a comparative overview of the main advantages and disadvantages of the different waste management strategies for fiber-reinforced polymers (FRPs) based on recent studies, and Table 2 shows a comparison of glass fiber composite (GF) recycling methods in terms of the efficiency, mechanical strength and energy demand.

## 3. Reuse of GFRP in Cementitious Materials

The reuse of recycled glass fiber-reinforced polymers (rGFRPs) in cement-based materials is interesting since it presents several advantages. For this, mechanical recycling is the most appropriate method due to its ease of execution and the wide range of possibilities. rGFRPs are used either to replace fine particles (cement and/or sand) or as reinforcement. Numerous studies have been conducted using rGFRP powder or particles (clusters) as a substitute for cement and/or aggregate, or as recycled fibers for reinforcement and crack control [8,43]. The particle size (including the fiber length) and shape are the key parameters that condition their performance in cement-based materials (Table 3). It should be noted that rGFRPs were already being used in rotary kilns for cement production. However, this method is tending to be limited due to some problems such as the lower quality of clinker due to impurities and management of toxic gases in kiln chimneys.

### 3.1. rGFRP Reaction with Cementitious Matrices

When added to a cementitious matrix, rGFRP particles dissolve and react with the surrounding environment. This reaction mechanism depends on the composition of the rGFRP (type of resin, glass fiber) as well as the pH of the medium. The dissolution process appears to be quite complex and may be (i) congruent (ion release controlled by surface/stoichiometry), (ii) incongruent (controlled by diffusion/leaching/selective/non-stoichiometric release), or (iii) a mixed dissolution involving both selective and congruent behavior [44]. Depending on the components released during dissolution, reactions may occur that can delay hydration and lead to the formation of gels.

First, the resin in the concrete environment may lower the pH by triggering reactions that consume hydroxyl ions through a series of chemical processes, forming thiolate, carboxylate, and alcohol ions. This pH reduction has a retarding effect on the cement hydration process [44].

GF in wind turbine blade waste is composed of SiO_2_, CaO, Al_2_O_3_, and minor traces of MgO and Na_2_O, and it could be a significant source of silica upon dissolution. In a high-pH environment, hydroxyl ions (OH^−^) progressively attack the ≡Si–O– bonds, leading to the gradual dissolution of the silica network. Consequently, Ca^2+^ supplied by the cement can react with the dissolved silica (and alumina) to form C-(A)-S-H gels [44,45,46].

### 3.2. Benefits and Limits of the Incorporation of rGFRP in Cementitious Materials

#### 3.2.1. Benefits

**Shrinkage reduction**

Water-induced shrinkage of cementitious materials is caused by capillary depressions that create internal stresses leading to the contraction of the material. Depending on the origin of the capillary depressions (water consumption by cement hydration, evaporation due to drying, water absorption by aggregates, etc.), there are several types of shrinkage (autogenous shrinkage, drying shrinkage, etc.). In addition, a distinction is made between plastic shrinkage occurring when the material has not yet hardened and shrinkage in the hardened state. The development of capillary stresses within the pore structure can result in the formation of cracks, especially when the strains are restrained by the boundary conditions or at the transition plastic/hardened state. Various solutions exist to attenuate this risk of early-age cracking, including the incorporation of glass fibers [47].

**Figure 6:**
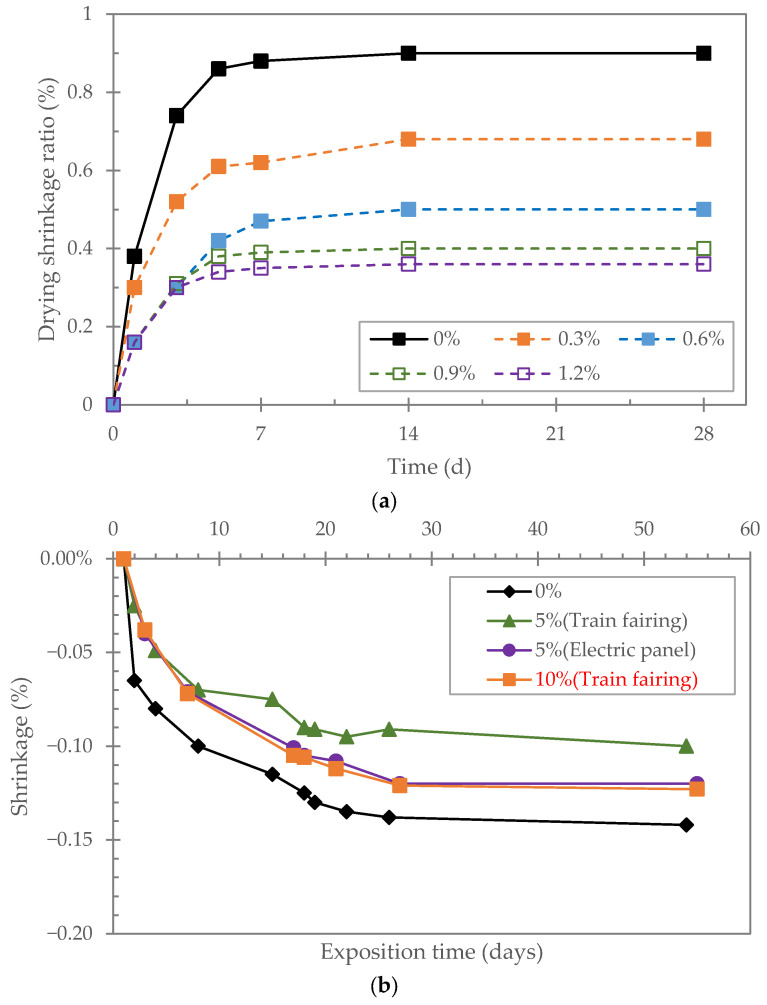
Effect of different rGFRPs on the drying shrinkage of mortar. (**a**) Glass fiber as reinforcement in MSH mortar (adapted from [54]). (**b**) Different origin of rGFRP that replaced sand (adapted from [55]).

The reuse of glass fibers recovered from recycled GFRP could be a cost-effective and sustainable solution to control plastic shrinkage cracking. Several studies have demonstrated the positive effect of recycled glass fibers on shrinkage [8,48,49,50] (Figure 6). For instance, Tao and Hadigheh [51] found that the incorporation of 5% of rGFRP (microfibers) significantly reduced the drying shrinkage, while the incorporation of 10% of rGFRP had only a limited effect. Zhou et al. [50] reported the same conclusion with 10% of rGFRP, as did Dehghan et al. [52]. Zhang et al. [53] found that the incorporation of rGFRP in concrete significantly reduces the early shrinkage (up to 66.5% compared to the control sample) and effectively prevents shrinkage cracking. The optimal fiber content was 7.5 kg/m^3^. However, it seems that the positive effect (limiting the shrinkage) decreases with excessive rGFRP content [53].

**Crack control and toughness enhancement**

**Figure 7:**
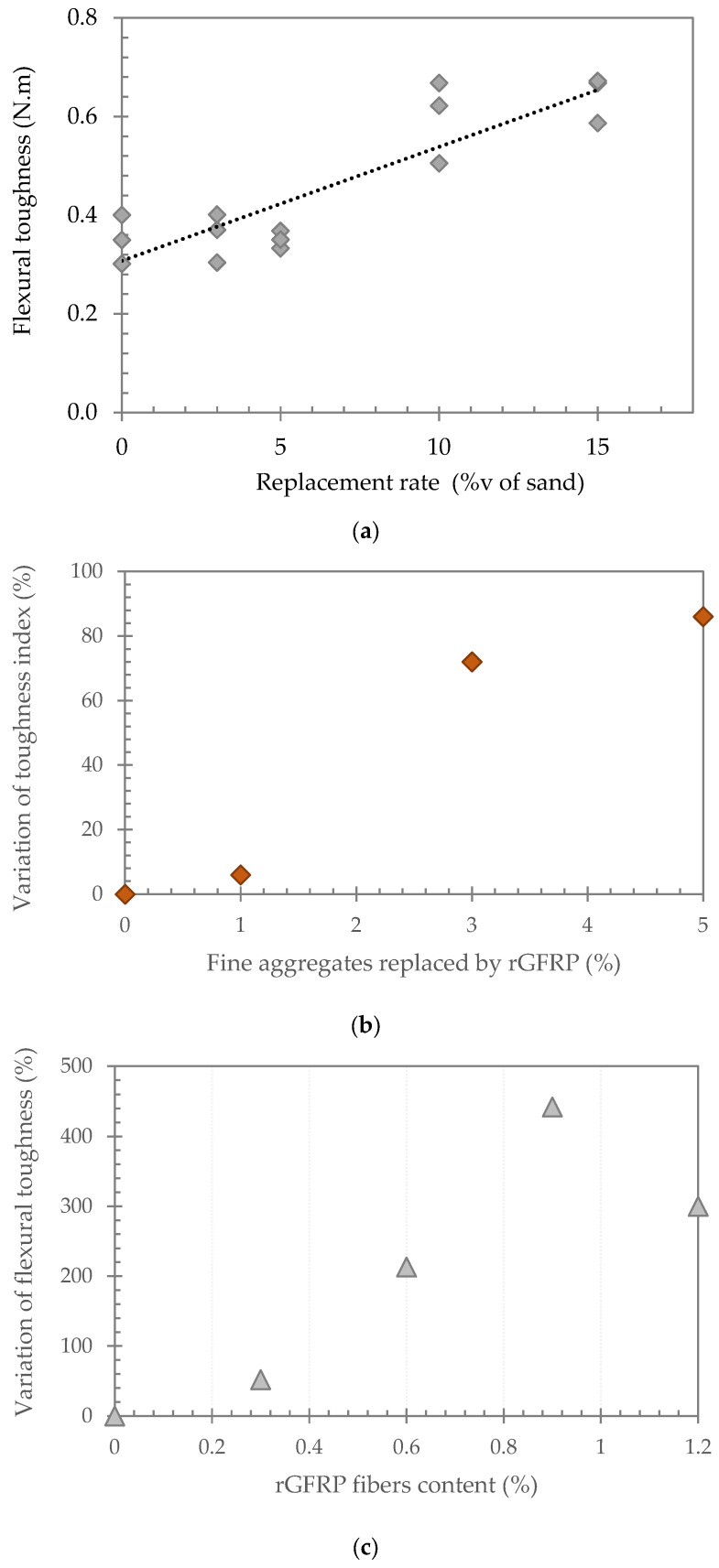
Effect of rGFRP on the flexural toughness of cementitious materials and impact resistance: (**a**) rGFRP powder 0/2 mm replacing sand [7]; (**b**) rGFRP large particles replacing sand [56]; and (**c**) glass fiber-reinforced [54].

The incorporation of rGFRP can contribute to controlling the development of cracks and improving the flexural toughness [7,56] (Figure 7). The substitution of 1% to 3% of volume of sand by rGFRP in mortar samples resulted in a larger post-peak area in the load–deflection curve, i.e., a larger strain energy [2]. For large rGFRP particles with a low volume content (1 and 2%), a sudden drop in the charge after the peak was observed by Haider et al. [2]. This indicates poor load transfer from the matrix to the rGFRP after matrix cracking. This behavior is attributed to the insufficient amount of rGFRP reinforcement in the tensile zones and at crack locations, combined with the fiber alignment along the load direction and the strong fiber–matrix adhesion, which enables effective load transfer. Longer fibers can fill macrocracks and contribute more to post-peak ductility [2,7].

The partial replacement of sand with varying proportions of rGFRP, ranging from 3% to 15% by sand volume [16], resulted in a more gradual load reduction compared to the abrupt failure observed in the reference mortar, as well as slower crack propagation in the rGFRP-modified mortars, consistent with findings from other studies. The toughness energy, toughness indices, and residual strength increase progressively with both the amount of incorporated rGFRP and the particle size [2,7]. Large rGFRP, when substituted in 5% and 7% volume of sand by rGFRP showed exceptionally higher toughness indices than 1, 2, and 3% volume substitution, with strain hardening behavior after the peak and a nearly 60% and 70% equivalent flexural ratio [2].

Thus, rGFRPs enable brittle fracture to be attenuated, reflecting an improvement in the toughness of cementitious materials incorporating rGFRPs, which contributes to a reduction in crack propagation that could be caused by the restrained shrinkage [2,7,56,57,58,59].

Furthermore, the incorporation of glass fibers into cement-based materials leads to an improvement in flexural strength (Figure 6) [8,43,55,60]. However, the effect of rGFRP on flexural strength can be different depending on several factors.

Mastali et al. [61] studied the impact of recycled glass fiber (0.25%, 0.75% and 1.25%vol) on flexural strength at 28 days of curing and found an increase of 30.1%, 42.9% and 59.5%, respectively, compared with the reference. The maximum flexural strength was reached at 1.25%vol.

Haider et al. [2] found that the incorporation of rGFRP particles as a partial replacement for sand up to 3% by volume improved the flexural strength. Rodin et al. [56] tested cement mortars incorporating 1, 3 and 5%vol replacement of sand by GFRP coming from wind turbines. It was shown that the flexural strength increased for large and small GFRPs, while the medium GFRP had a 6% reduction and GFRP powder resulted in a 30% reduction.

In the study carried out by Farinha et al. [43], replacing sand with rGFRP powder up to 50% by volume of sand resulted in an improvement in flexural strength up to 180 days of curing, depending on the amount of rGFRP. Asokan et al. [62] studied the effect of two different prototype architectural panels (300 mm × 300 mm × 8 mm and 300 mm × 300 mm × 12 mm) of cementitious composites using 5% rGFRP (by weight of cement content) on the flexural strength and found an increase in flexural strength of about 36% and 25%, respectively, for 12 mm and 8 mm thick panels reaching 16.5 MPa and 8.8 MPa. An improving effect was observed when rGFRP is incorporated as a fibrous reinforcement in concrete and mortar [50]. The effect of rGFRP on mechanical performance and particularly flexural strength may be explained by the bridging effect of the GFRP fibers that can partially transfer stress across the cracks [7,8]. This effect would be mainly governed by the length and the shape as well as the random distribution of rGFRPs [6,7,51,56].

Another interesting mechanical property that can be enhanced by the incorporation of rGFRP is the impact resistance. As shown by Mastali et al. [61], the incorporation of 0.25%, 0.75% and 1.25% (volume fraction) rGFRP increased the first crack impact resistance 2.53, 3.73 and 5.06 times, and the ultimate crack impact resistance increased 2.94, 4.44 and 6.14 times, respectively.

**Lightening and density reduction**

**Figure 8:**
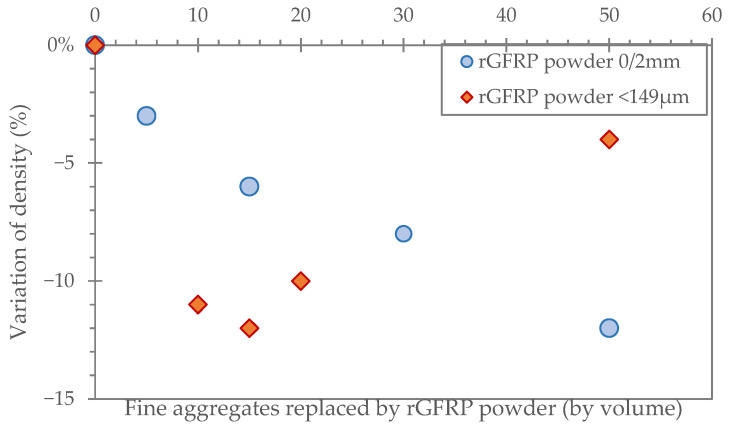
Effect on the density of rGFRP replacing fine aggregates: rGFRP powder 0/2 mm [62]; and rGFRP powder < 149 µm [43].

The hardened density can be reduced by the incorporation of rGFRP in mortars and concretes, depending on the size and dosage of the rGFRP, which is justified by the low density of rGFRP particles compared to natural aggregate particles [7,8,54,56,62,63,64] (Figure 8). Zhao et al. [65] investigated the effect of substituting 3% and 5% (by weight of sand) of fine particles in the concrete with rGFRP after 28 days of curing. They found a reference concrete density of 2.42 g/cm^3^ and a reduction in density of 1.74% and 3.93%, respectively, for 3% and 5% substitution. Kaboré et al. [6] substituted 10%vol of sand by different size fractions of rGFRP in mortar and found a significant reduction in the density of the mortars containing more fine particles of rGFRP, while the density reduction was lower when the particle size distribution of rGFRP was fairly homogeneous.

Although reducing the density makes the material lighter and lowers its own weight, which can be useful in some applications, it has to be kept in mind that this reduction is often accompanied by a decrease in mechanical strength, particularly compressive strength [6,66].

#### 3.2.2. Limits

**Workability reduction**

**Figure 9:**
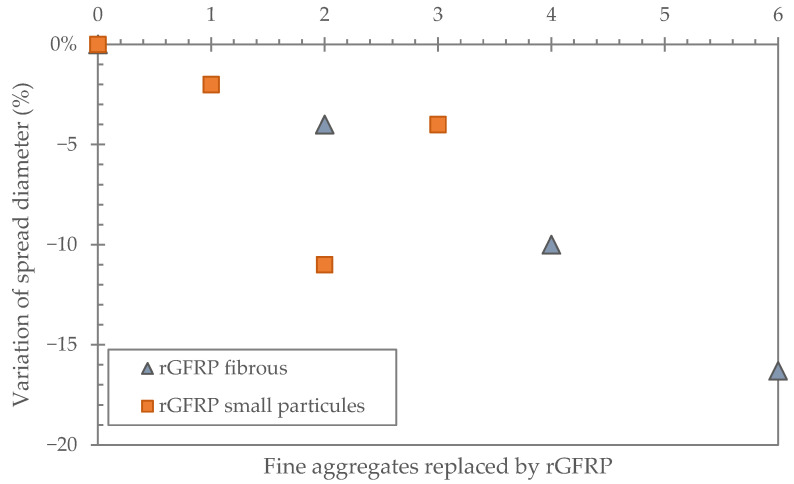
Effect of rGFRP on the workability: rGFRP fibrous [50]; and rGFRP small particle [2].

Referring to the ability of materials to be easily mixed and placed, workability is the primary factor to consider for cement-based materials, as it is an essential property directly affecting the implementation and final durability of cement materials. It depends on several phenomena and can be adjusted with the amount of water that the addition of would negatively impact mechanical performance. The incorporation of rGFRP in general has a negative impact on workability [7,48,67,68] (Figure 9). This negative effect can be explained by the higher water absorption of rGFRP, which increases the water demand of the mixture and thus reduces the workability [43,69]. El Bitouri et al. [7] reported an increase in the water required to maintain the workability with the replacement rate of sand by rGFRP.

Zhao et al. [65] studied the effect of different types of rGFRP (powder and cluster) as a substitute for sand on the workability (slump) of concrete. The results showed a decrease in slump with increasing amounts of rGFRP. Moreover, rGFRP powder decreased the slump more than rGFRP clusters, so the rGFRP size influences workability [65]. Other authors found the same results and attributed it to the flocculation of particles (or fibers), which increases the water demand [55,57]. The agglomeration of rGFRP with irregular shape and size and the higher specific surface area thereby increase the water demand [30,65]. Thus, the reduction in available water for cement particles hinders their hydration, limiting the formation of portlandite and consequently the development of gels such as C-S-H, which affects long-term strength. Haider et al. [2] substituted 1% to 5% by volume of sand with different sizes of rGFRP and found no significant impact on the workability of the mortar. Only at 5% was a decrease in flow diameter of 18% observed compared to raw concrete without rGFRP. As concluded by some authors, a low addition of rGFRP does not have a significant effect on workability but leads to an acceptable slump or spread diameter of concrete and mortar [7,8,57,61].

Kaboré et al. [6] showed that the intensity of the negative impact of rGFRP on workability can be reduced by changing the order in which the materials are mixed. They first mixed rGFRP particles with water to release the water trapped in flocculated particles. This approach makes it possible to produce mortar without adjusting the water/cement ratio, and therefore, to limit the loss of compressive strength.

**Reduction of mechanical strength**

**Figure 10:**
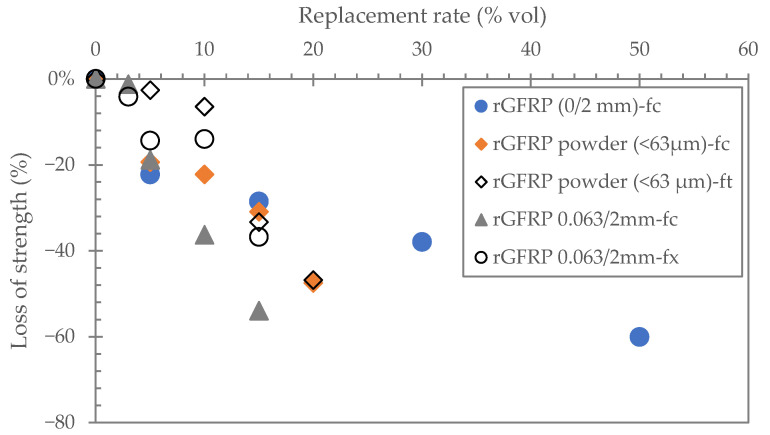
Effect of rGFRP on the mechanical strength of cementitious materials at 28 days of curing, as a function of the fine aggregate replacement (% by volume). fc: compressive strength, ft: tensile strength, fx: flexural strength (rGFRP 0/2 mm [62]; rGFRP powder < 63 µm [68], rGFRP 0.063/2 mm [7]).

Several studies have reported a decrease in compressive strength with the incorporation of rGFRP. Rodin et al. [56] found a decrease in compressive strength of 26% to 41% compared to the control by replacing 3% by volume of sand with small and medium-sized rGFRP particles (Figure 7). Zhou et al. [70] replaced concrete aggregates (30, 50, 70 and 100% by volume) with coarse rGFRP. A decrease in compressive strength was observed depending on the number of substitutions (Figure 10).

Baturkin et al. [71] reported that the incorporation of rGFRP as powder caused a significant reduction in the compressive strength, mainly due to the presence of organic content interfering with cement hydration, while the inclusion of rGFRP as fiber reinforcement resulted in an enhancement in flexural strength without a noticeable reduction in compressive strength. The incorporation of rGFRP in the form of aggregates resulted in a significant decrease in compressive strength due to the lower adherence with the matrix [71].

El Bitouri et al. [7] replaced 3%, 5%, 10% and 15% by volume of sand with rGFRP in mortar and observed no significant decrease in compressive strength at 3%, but from 5% and more, a significant reduction was observed, reaching 54% reduction at 15%vol of rGFRP. This reduction in compressive strength is mainly due to the adjustment of the water to binder ratio to maintain workability. The pre-mixing of rGFRP with water before adding cement and sand allowed for the workability to be maintained without increasing the water content and thus decreasing the loss of strength [6]. In addition, Kaboré et al. [6] showed that the addition of rGFRP in the form of fiber/powder mix (0/2 mm) in substitution for 10%vol of sand resulted in a slight improvement in flexural strength without a significant decrease in compressive strength.

Yazdanbakhsh et al. [59,72] replaced 40% and 100% of coarse aggregates with GFRP rebars waste in concrete and observed a decrease in compressive strength by 13% and 21%, respectively. Zhou et al. [50] studied the effect of mortar reinforcement with two types of rGFRP, one raw and the other cleaned (careful hydraulic and aerodynamic selection containing only fibrous fraction), and also other variable factors, including the size and different surface contact angles, at addition rates of 2%, 4% and 6%. They observed firstly a decrease in compressive strength with raw rGFRP, then for cleaned rGFRP, their incorporation resulted in an improvement in compressive strength depending on the surface contact angle.

The decrease in mechanical strength (particularly compressive strength) can be attributed to several factors, including firstly the compressive strength of rGFRP being lower than that of sand particles and aggregates. Secondly, an increase in voids (porosity) due to air entrapment and poor particle arrangement and distribution (and water to binder adjustment), and finally, weaker interfacial bonding between rGFRP particles and the cement matrix due to the smooth cylindrical shape and size of rGFRP particles [6,7,8,48,59,63,68,72,73].

For instance, substituting 10%vol of sand by different size fractions of rGFRP (0.063 mm to 2 mm) resulted in an increase in porosity of about 11% after 7 months of curing [6]. Farinha et al. [43] observed a decrease in the open porosity of mortar containing up to 15%vol replacement of sand by rGFRP powder, while for higher GFRP contents, the open porosity increased. Farinha et al. [43] attributed this decrease to the filling effect induced by rGFRP particles. Oliveira et al. [63] reported an increase in voids from 27% to 47% for a sand substitution by mass with rGFRP from 7.5% to 37.5%. The incorporation of recycled GFRP fibers had very little impact (both a small increase and decrease depending on the type of rGFRP) on the pore volume [50]. The incorporation of rGFRP powder decreases the average pore size but induces a wide pore-size distribution due to the air entrapment (larger voids) and filler effect (small voids) [8,63,74]. The incorporation of rGFRP, which increases the void content, also weakens the interfacial transition zone (ITZ) between the waste particles and the binding matrix [44].

**Risk of alkali–silica reaction (ASR)**

Glass fibers contain silica and may trigger alkali–silica reactions (ASRs) in alkaline cementitious environments, potentially compromising durability. Rodin et al. [56] studied the risk of alkali–silica reactions in mortar containing rGFRP particles and observed expansion, but which did not reach the critical threshold. The risk appears limited but warrants further investigation, particularly for long-term durability assessments. Dehghan et al. [52] studied the expansion of mortar and concrete by replacing 5% by mass of crushed aggregates and fine particles with different types of rGFRP (shape, size). The expansion values for all the mortar mixtures produced were below the recommended limit of 0.1%. Furthermore, the test results on concrete prisms with different types of recycled GFRP showed no particular expansion values and were all below the recommended expansion limit of 0.4%. Several factors may explain the inhibition of ASR. (i) The type and origin of the glass fibers, since E-glass fibers differ from conventional amorphous glass (e.g., bottle glass) and are less reactive to alkalis. Additionally, the particle size has a significant impact, as coarse particles have a lower specific surface area and are therefore less reactive [75,76]. (ii) The resin coating the glass fibers limits their contact with the alkaline environment and the occurrence of reactions. (iii) A decrease in pH caused by components of the resin may also reduce ASR activity. According to Tittarelli et al. [49], studies on the alkali–silica reactivity (ASR) of ground glass fiber-reinforced polymer (GFRP) show that replacing part of the fine aggregates with GFRP powder does not negatively affect the durability of Portland cement concrete and mortar, thereby excluding any potentially harmful reactivity [71].

**Table 3:** Comparison of the impact of the shape and quantity of rGFRP on the performance of cementitious materials.

Reference	Quantity of rGFRP	Length/Size	Shape	Materials Replaced	Compressive Strength	Flexural Strength	Toughness	Porosity	Shrinkage
El Bitouri et al. [7]	0%; 3%; 5%; 10%; 15%	0.8 mm–2 mm diameter	Mix of powder, particles and small fiber of rGFRP	Sand	Down to 54%	Down to 27%	Up to 83%	-	-
Haider et al. [2]	0%; 1%; 2%; 3%	Different lengths 0.51 mm–25 mm 1.07 mm–34.6 mm 8.88 mm–70.45 mm		Sand	Up to 13%	Up to 85%	-	-	-
Kaboré et al. [6]	10%	0.063 mm–2 mm diameter	Mix of powder, particles and small fiber of rGFRP	Sand	Down to 31%	Down to 9.6% and Up to 2.72%	-	Up to −15%	-
Oliveira et al. [63]	0%; 7.5%; 12.5%; 15%; 30.5%; 37.5%	0.5 mm–3 mm diameter	Powder	Sand	Down around 70%	Down around 30%	-	Increase in voids until 47%	-
Rodin et al. [56]	-	<0.42 mm 0.42 mm–0.841 mm 1.41 mm–2 mm 2 mm–2.38 mm	Powder, small, medium and large size of rGFRP	Sand	Down to 65%	Up to 60%	Up to 86%	-	-
Yazdanbakhsh et al. [72]	40%; 100%	9.5 mm–25 mm	Coarse cylindric aggregates of rGFRP	Aggregates	Down to 13% and Down to 21%	-	-	-	-
Zhao et al. [65]	1%; 3%; 5%	<1.18 mm 1.18 mm–8 mm	Powder and cluster of rGFRP	Fine Aggregates	Down to 11% and Up to 7%	Down to 9% and Up to 4	-	-	-
Baturkin et al. [71]	10%; 20%; 30%; 33%; 66%; 100%	-	Powder and particles of rGFRP	Cement and Aggregates	Down to 67% and Down to 72%	Down to 27% and Down to 57%	-	-	-
REINFORCEMENT									
Baturkin et al. [71]	1%; 1.75%	-	Fiber of rGFRP	-	Down to 4%	Up to 15%	-	-	-
Mastali et al. [61]	-	20 mm length 0.11 mm diameter	Fiber of rGFRP	-	Up to 50%	Up to 23%	Crack impact up to 59.5%	-	-
Zhou et al. [50]	2%; 4%; 6%	0.3 mm–5.3 mm diameter 0.8 mm–23.4 length	Powder and fiber cluster of rGFRP	-	Down to 15%	Up to 21%	-	Increasing pore size (0.5 mm–1.7 mm)	Down to 23%

## 4. Potential Applications of Cementitious Materials Containing rGFRP

Fiber-reinforced cementitious materials are widely used in construction to improve specific properties for targeted applications. The incorporation of rGFRP into mortars and concretes demonstrates promising performance in several areas, suggesting its potential use in a wide range of applications as a cost-effective and sustainable solution.

**Figure 11:**
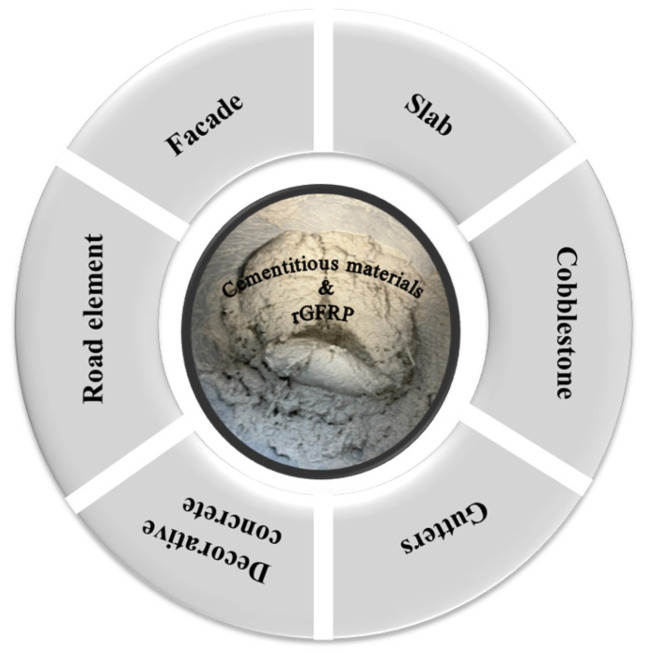
Field of application of cementitious materials containing rGFRP.

Since compressive strength tends to decrease with increasing rGFRP content [2,6,7], these materials are not recommended for load-bearing structural elements. However, their enhanced flexural strength, toughness, impact resistance and shrinkage control make them suitable for a variety of other uses (Figure 11).

Firstly, rGFRP-enhanced mortars and concretes could be applied as floor toppings, screeds, or thin slabs, where improved cracking resistance and reduced shrinkage are advantageous (Figure 8). These properties may reduce or eliminate the need for expansion joints, while also improving impact resistance.

Secondly, due to their ability to limit cracking, rGFRP-modified materials may be used for rendering mortars, facade coatings and prefabricated facade panels. Their light weight also offers benefits for prefabricated construction.

Additional potential applications include decorative concrete, paving stones, drainage channels and elements used in public works and landscaping. In the road construction sector, these materials could be used for sidewalks, curbs, gutters and signage elements.

One key advantage of rGFRP lies in its dual functionality: it can act both as a reinforcing fiber and as a partial replacement for natural sand. This contributes to reducing the consumption of natural aggregates and, in turn, the environmental impact of cementitious materials.

Following the beneficial effect of rGFRP on the properties of cementitious materials, the application domains could be defined. However, a large-scale study on full-size samples is necessary after small-scale characterizations, where new factors must be considered that could negatively impact the properties. At full scale, certain issues may arise, such as more difficult control of mixture homogeneity and variations in implementation conditions depending on weather conditions.

## 5. Challenge of Reusing GFRP in Cementitious Materials

The recycling of GFRP composites in cementitious materials may face competition within this field of valorization. Indeed, materials such as end-of-life plastics and rubber are already commonly reused in mortars and concretes. These waste materials offer similar improvements in terms of the mechanical properties. Moreover, they benefit from an established market presence and greater availability. However, the introduction of rGFRP would expand the range of available materials, and combining them with other types of waste could potentially lead to enhanced overall performance in cementitious materials. A study found that replacing 10% sand by volume with recycled plastic is a viable proposition that has the potential to save 820 million tons of sand every year [77]. The addition of GFRP waste as a recyclable material in cementitious composites could increase the volume of recoverable waste and help reduce sand consumption in the construction sector.

## 6. Perspectives and Recommendations

Although numerous studies have been devoted to the integration of recycled GFRP into cementitious matrices, several knowledge gaps remain. Most research has focused on the impact of recycled GFRP particles on the workability, mechanical properties, density, and porosity of concrete and mortar containing these materials. However, the microstructural analysis of these composites is still underexplored, and the chemical interactions between rGFRP particles and cement particles have rarely been addressed. Moreover, the determination of the optimal size and length of rGFRP particles, which could play a major role in enhancing performance, remains largely unexplored.

To make the recycling of GFRP in cementitious materials truly viable, it is also necessary to conduct long-term durability studies, particularly under environmental exposure conditions. Assessing the behavior of these materials under freeze–thaw cycles, humidity, and other aggressive agents is essential to ensure their structural reliability. Furthermore, the potential environmental impacts of these recycled materials (on air, water, and soil) must be rigorously investigated. Finally, a comprehensive techno-economic analysis is crucial to evaluate the industrial feasibility and economic viability of this recycling pathway.

## 7. Conclusions

This paper presents a brief but comprehensive review of the possibility of reusing GFRP in cementitious materials, providing information on its impact on various key characteristics. Reusing recycled GFRP in cement-based materials is an encouraging and viable option.

Among recycling methods, mechanical recycling appears to be the most economical, with less impact on the environment. It is also the most compatible with cementitious applications, as it enables the incorporation of both fiber and resin.

The procedure used for mechanical recycling (comminution process) is a major factor affecting the size, shape and fiber/resin fraction of rGFRP.

The addition of rGFRP generally reduces workability. However, this effect depends on several factors, such as the particle shape, size and the method of incorporation. Proper mix design adjustments can help mitigate this drawback.

The substitution of natural aggregates by rGFRP reduces the density but often increases the porosity, which negatively affects the mechanical strength and durability. Compressive strength tends to decrease with increasing rGFRP content. Conversely, flexural strength is generally less affected and can sometimes increase depending on the shape, length and content of the recycled GFRP.

Notably, the incorporation of rGFRP improves toughness and reduces crack propagation. These enhancements are particularly linked to the bridging action of fibers, which can be very useful in limiting the effect of restrained shrinkage.

Although glass fibers are not inherently alkali-resistant, they appear to remain well preserved within the alkaline cement matrix, likely due to the protective resin layer.

## Figures and Tables

**Table 1:** Comparative overview of the advantages and disadvantages of different FRP waste management strategies [16,17,19,22].

Management Route	Advantages	Disadvantages	Essential Points
Landfill	Cost-effective Easy to install	Pollution of the local environment Greenhouse gas emissions (methane) Accumulation of toxic waste that causing health issues	Government legislation prohibiting the installation Limited space
Incineration	With or without energy recovery Efficient use of space and reduced space required for installation	No material recovered 50% of waste turns to ash Expensive to build, operation and maintenance Atmospheric pollution from combustion gases	Non-recovery method 50–70% remains as ash Entry fee charged
Incineration or processing in cement kiln	Recovery of materials and energy Use of FRP waste as primary or supplementary fuel Highly efficient, fast and scalable No ash or residue Transformation of GFRP waste into energy and clinker	Loss of original fiber shape Additional energy required to reach high processing temperatures Reduction of GFRP into smaller sizes before processing	Only suitable for GFRP Emissions of pollutants and particulates
Mechanical recycling	Efficient, scalable to industrial scale No air or water Low-cost equipment, no skilled labor required	Health and safety problems (risk of ignition and dust exposure) Low-value recyclates competing poorly with virgin materials	No recovery of individual fibers Produces low-value products (only for GFRP) Requires enclosed facilities area to limit dust
Thermal recycling: pyrolysis	By-products (gas, oil) can be used as energy Easily scalable Already used commercially for carbon fiber recycling	Fibers may retain oxidation or carbon residues Lower-quality recovered fibers Fiber strength loss due to high temperatures Not always economically viable	Potential gas leakage from treatment chambers
Chemical recycling: solvolysis	Recovery of clean, long fibers Recovery of reusable resins Use of relatively safe solvents (alcohols, glycols, supercritical water)	Low efficiency and high cost High energy demand (temperature and pressure) Large solvent volumes required	Potential greenhouse gas impacts

**Table 2:** Comparison of glass fiber composite (GF) recycling methods in terms of the efficiency, mechanical strength and energy demand [5,21,41,42].

Recycling Technique	Energy Demand MJ/kg	Fiber Recovery Rate (%)	Tensile Strength Maintained (%)
Mechanical	0.4–4.8	58–89	79
Pyrolysis	10–36	55–83	35–80
Fluidized Bed	30–40	66	50
Solvolysis	26–91	45–95	31–58

## Data Availability

No new data were created or analyzed in this study.
